# Nontubular epithelial Na^+^ channel proteins in cardiovascular regulation

**DOI:** 10.14814/phy2.12404

**Published:** 2015-05-19

**Authors:** Heather A Drummond

**Affiliations:** Department of Physiology and Biophysics, University of Mississippi MedicalJackson, Mississippi, USA

The role of Epithelial Na^+^ Channel (ENaC) proteins in cardiovascular regulation and blood pressure control traditionally have been viewed through the lens of ENaC-dependent salt and water transport in the renal cortical collecting duct. In this nephron segment, decreases in ENaC expression/activity lead to salt and water wasting resulting in hypotension and increases lead to hypertension via increased renal salt and water reabsorption. However, the article entitled “Enhanced expression of epithelial sodium channels causes salt-induced hypertension in mice through inhibition of the *α*_2_ isoform of Na^+^, K^+^ ATPase” in the current issue (Leenen et al. 2015), as well as previous publications from this and other groups, demonstrate that non-tubular ENaC proteins contribute to cardiovascular regulation beyond their direct role in cortical collecting duct salt and water transport (Huang and Leenen 2005; Guan et al. 2009; Wang et al. 2009; Kusche-Vihrog et al. 2010; Drummond 2012).

At least three nontubular ENaC roles contribute to cardiovascular regulation. First, there is the role of central ENaC proteins. ENaC in the choroid plexus and cardiovascular-regulatory brainstem nuclei, as partially addressed in the publication noted above, sense increases in cerebral spinal fluid Na^+^ concentration and in response, increase sympathetic nerve activity to induce peripheral vasoconstriction and proximal tubule Na^+^ transport (Huang and Leenen 2005).

The second nontubular role for ENaC relates to endothelial cell function. Similar to cortical collecting duct ENaC, endothelial cell ENaC is gated by shear stress, a stimulus known to induce nitric oxide release and vasodilation (Satlin et al. 2001; Wang et al. 2009). ENaC also influences endothelial cell membrane stiffness-dependent nitric oxide release (Kusche-Vihrog et al. 2010). How ENaC mediated shear stress and endothelial membrane stiffness interact to control vascular tone has yet to be resolved. However, this ENaC-dependent response represents a novel pathway for regulation of vascular tone and warrants further investigation.

The third nontubular role of ENaC is in vascular smooth muscle cell (VSMC) mediated pressure-induced, or myogenic, constriction. This response is inherent to small arteries and arterioles in the kidney, brain, gut, skeletal muscle, and heart and functions independent of neural influences. My laboratory and others have shown that the mammalian ENaC proteins also function as mechanosensors in vascular smooth muscle cells (VSMCs) that initiate the response. The myogenic response serves as a mechanism of renal blood flow autoregulation and offers protection to microvessels from higher systemic pressure. Consistent with these functions, mice with reduced levels of *β*ENaC have the following alterations: (1) abolished (~90%) myogenic constriction in renal afferent arterioles, (2) attenuated (~50%) myogenic regulation of renal blood flow, (3) signs of mild renal injury, and (4) elevated blood pressure (Drummond 2012). The increased blood pressure is not likely due to loss of tubular ENaC, as that would favor salt/water loss and, thus, hypotension. When viewed thru the lens of cortical collecting duct salt/water transport, the elevated blood pressure in the reduced *β*ENaC model may seem counterintuitive; however, it is consistent with the role of ENaC proteins as mediators of the renal-protective myogenic response.

These three nontubular roles of ENaC proteins share two common features. First, the signaling mechanisms converge on a final common pathway for control of blood pressure; long-term regulation of renal Na^+^ and water balance. Activation of central ENaC by changes in local [Na^+^] stimulation of sympathetic nerve activity should increase renal vascular resistance and enhance Na^+^ reabsorption at several sites along the nephron and stimulate renin/angiotensin II/aldosterone signaling with additive effects on renal hemodynamics and salt/water transport. Shear stress-mediated vasodilation and myogenic-mediated vasoconstriction should also influence renal vascular resistance. Increases in renal vascular resistance increase renal tubular Na^+^/water transport by changes in peritubular capillary pressure. In the long term, loss of myogenic constriction can cause renal injury, which is linked to hypertension through nephron loss. Thus, the non-tubular actions of ENaC also contribute to the long-term balance of renal salt and water, and thus blood pressure homeostasis. Second, the ENaC proteins appear to function as sensors of the extracellular environment, whether it is extracellular [Na^+^], shear stress or strain. The closely related nematode degenerin and mammalian Acid Sensing Ion Channel (ASIC) proteins, also appear to function as extracellular proton and/or mechanosensors (Kellenberger and Schild 2002). ENaC/ASIC protein structure is ideally suited for an environmental sensor. Based on the crystal structure of ASIC1, these proteins have a single large extracellular domain (~400 residues) that is shaped like a fist and rises above the plasma membrane approximately 90 Å (Jasti et al. 2007). This is much larger than the Transient Receptor Potential (Trp) family of proteins, another large protein family linked to mechanosensing. Most Trp family members have ~100 residues distributed across 4 extracellular domains (Montell 2005). Thus, the extracellular domain of ENaC/ASIC proteins is ideal to interact with extracellular milieu of ions, growth/autocrine/paracrine factors and matrix proteins to regulate and tune its sensitivity to extracellular factors.

It is time to view the role of ENaC proteins in the integrative control of blood pressure through several lens, including the direct effect on cortical collecting duct Na^+^/water reabsorption, and its indirect roles in the central control of sympathetic nerve activity, shear stress and myogenic regulation of vascular tone.
